# Quantitative Molecular Detection of 19 Major Pathogens in the Interdental Biofilm of Periodontally Healthy Young Adults

**DOI:** 10.3389/fmicb.2016.00840

**Published:** 2016-06-02

**Authors:** Florence Carrouel, Stéphane Viennot, Julie Santamaria, Philippe Veber, Denis Bourgeois

**Affiliations:** ^1^Institute of Functional Genomics of Lyon, UMR CNRS 5242, Ecole Normale Supérieure de Lyon, University Lyon 1, LyonFrance; ^2^Laboratory “Health, Individual, Society” EA4129, University Lyon 1, LyonFrance; ^3^Department of Prevention and Public Health, Faculty of Dentistry, University Lyon 1, LyonFrance; ^4^Laboratory “Biométrie et Biologie Évolutive”, UMR CNRS 5558 – LBBE, University Lyon 1, VilleurbanneFrance

**Keywords:** oral biofilm, periodontology, Socransky complexes, *P. gingivalis*, interdental microbiota

## Abstract

In oral health, the interdental spaces are a real ecological niche for which the body has few or no alternative defenses and where the traditional daily methods for control by disrupting biofilm are not adequate. The interdental spaces are the source of many hypotheses regarding their potential associations with and/or causes of cardiovascular disease, diabetes, chronic kidney disease, degenerative disease, and depression. This PCR study is the first to describe the interdental microbiota in healthy adults aged 18–35 years-old with reference to the Socransky complexes. The complexes tended to reflect microbial succession events in developing dental biofilms. Early colonizers included members of the yellow, green, and purple complexes. The orange complex bacteria generally appear after the early colonizers and include many putative periodontal pathogens, such as *Fusobacterium nucleatum*. The red complex (*Porphyromonas gingivalis*, *Tannerella forsythia*, and *Treponema denticola*) was considered the climax community and is on the list of putative periodontal pathogens. The 19 major periodontal pathogens tested were expressed at various levels. *F. nucleatum* was the most abundant species, and the least abundant were *Actinomyces viscosus, P. gingivalis*, and *Aggregatibacter actinomycetemcomitans.* The genome counts for *Eikenella corrodens, Campylobacter concisus, Campylobacter rectus, T. denticola*, and *Tannerella forsythensis* increased significantly with subject age. The study highlights the observation that bacteria from the yellow complex (*Streptococcus spp*., *S. mitis*), the green complex (*E. corrodens, Campylobacter gracilis, Capnocytophaga ochracea, Capnocytophaga sputigena, A. actinomycetemcomitans*), the purple complex (*Veillonella parvula*, *Actinomyces odontolyticus*) and the blue complex (*A. viscosus*) are correlated. Concerning the orange complex, *F. nucleatum* is the most abundant species in interdental biofilm. The red complex, which is recognized as the most important pathogen in adult periodontal disease, represents 8.08% of the 19 bacteria analyzed. *P. gingivalis* was detected in 19% of healthy subjects and represents 0.02% of the interdental biofilm. *T. forsythensis* and *T. denticola* (0.02 and 0.04% of the interdental biofilm) were detected in 93 and 49% of healthy subjects, respectively. The effective presence of periodontal pathogens is a strong indicator of the need to develop new methods for disrupting interdental biofilm in daily oral hygiene.

## Introduction

Of all of the parts of the human body, the interdental (ID) space is a unique place, a real ecological niche, for which the body has few or no alternative defenses and where the traditional daily methods for control by disrupting biofilm are not adequate. This niche is the source of many hypotheses of its potential associations with and/or causes of cardiovascular disease (Teles and Wang, 2011; Desvarieux et al., 2013; Li et al., 2014; Boillot et al., 2015; Chistiakov et al., 2016), diabetes (Gomes-Filho et al., 2015), chronic kidney disease (Chambrone et al., 2013), obesity (Suresh and Mahendra, 2014), degenerative diseases (Jimenez et al., 2009), depression (Hsu et al., 2015), and premature labor (Corbella et al., 2015; Vanterpool et al., 2016). These hypotheses have become more forceful in publication after publication, despite the considerable differences between the studies (Simpson et al., 2010; Zi et al., 2015; Araújo et al., 2016).

For the mouth, the Human Oral Microbiome Database lists 1,200 predominant oral species, with some 19,000 phylotypes (Keijser et al., 2008), and with distinct subsets predominating in different habitats (Xu et al., 2015).

Due to its unusual anatomy between adjacent teeth and the gingival tissue, the interdental space – in fact the 30 interdental spaces for an individual – is a huge source of bacteria (Cafiero and Matarasso, 2013). This space favors the development of periodontal diseases in adults because the access required to disrupt the biofilm by toothbrushing and saliva is restricted (Dentino et al., 2013).

Periodontitis affects millions of people each year (Darveau, 2010). Periodontitis is considered a polymicrobial inflammatory disease and is characterized at its onset by a synergistic and dysbiotic microbiota, within which different members or specific gene combinations fulfill distinct roles that converge to shape and stabilize a disease-provoking microbiota (Darveau, 2010; Hajishengallis et al., 2012). Dysbiotic oral bacterial communities have a critical role in the etiology and progression of periodontal diseases (Mason et al., 2015) in the subgingival pockets (Kumar et al., 2005). However, these communities were described in the corona of the gingival margin (Uzel et al., 2011) and the salivary gland (Morozumi et al., 2016). It was also identified in the healthy supragingival plaque (Uzel et al., 2011).

An understanding of what is happening within these interdental spaces is a priority. This knowledge will lead to new recommendations and innovative preventive methods to significantly reduce gingivitis, periodontitis and related diseases. The first stage consists of qualitatively and quantitatively describing the microbiota of the interdental space from clinically healthy subjects. In the longer term, this information will enable the immune and inflammatory mechanisms involved in periodontal pathologies to be better understood. The objective of our bacterial cartography study is based on the benchmark framework of the Socransky complexes, which have the consensus of the scientific community (Socransky et al., 1998). Socransky et al. (1998) identified six complexes of bacteria that commonly occur together, and color-coded them as blue, green, yellow, purple, orange, and red. The blue, yellow, green, and purple complexes are compatible with periodontal health, whereas the orange and red complexes are correlated with periodontal disease (Socransky et al., 1998; Socransky and Haffajee, 2002).

Currently, no study has addressed interdental biofilm in healthy adults. The main objective of this study is to describe the interdental microbiota in healthy adults with reference to the Socransky complexes. For this purpose, a quantitative detection system has been developed that uses real-time PCR methodology to quantify 19 major periodontal pathogens, including the following bacteria from (i) the blue complex – *Actinomyces viscosus* (*A. viscosus, Av*); (ii) the purple complex – *Actinomyces odontolyticus* (*A. odontolyticus, Ao*) and *Veillonella parvula.(V. parvula, Vp)*; (iii) the green complex – *Aggregatibacter actinomycetemcomitans* (*A. actino. a*, *Aa*), *Campylobacter concisus* (*C. concisus, Cc), Capnocytophaga ochracea* (*C. ochracea, Co*), *Capnocytophaga sputigena* (*C. sputigena, Cs*), and *Eikenella corrodens* (*E. corrodens, Ec*); (iv) the yellow complex – *Streptococcus mitis (S. mitis)* and *Streptococcus spp*. *(Sspp);* (v) the orange complex – *Campylobacter gracilis* (*C. gracilis, Cg*), *Campylobacter rectus* (*C. rectus, Cr*), *Fusobacterium nucleatum* (*F. nuc, Fn*), *Parvimonas micra* (*P. micra*, *Pm*), *Prevotella intermedia* (*P. intermedia*, *Pi*), and *Prevotella nigrescens* (*P. nigrescens*, *Pn*); and (vi) the red complex -*Porphyromonas gingivalis* (*P. gingivalis, Pg*), *Tannerella forsythia* (*T. forsythia, Tf*), and *Treponema denticola* (*T. denticola, Td*).

Research findings can contribute greatly to improving the periodontal health of people. Findings can be used to make decisions on new policies in relation to molecular analysis about classification of periodontal diseases and provision of services (e.g., instituting new procedures, practices and interventions, including those for prevention) related to periodontal health-care delivery. Furthermore, research findings can also be used for advocacy or promoting the adoption of best practice to prevent or mitigate consequences of risks to health.

## Materials and Methods

**FIGURE 1:**
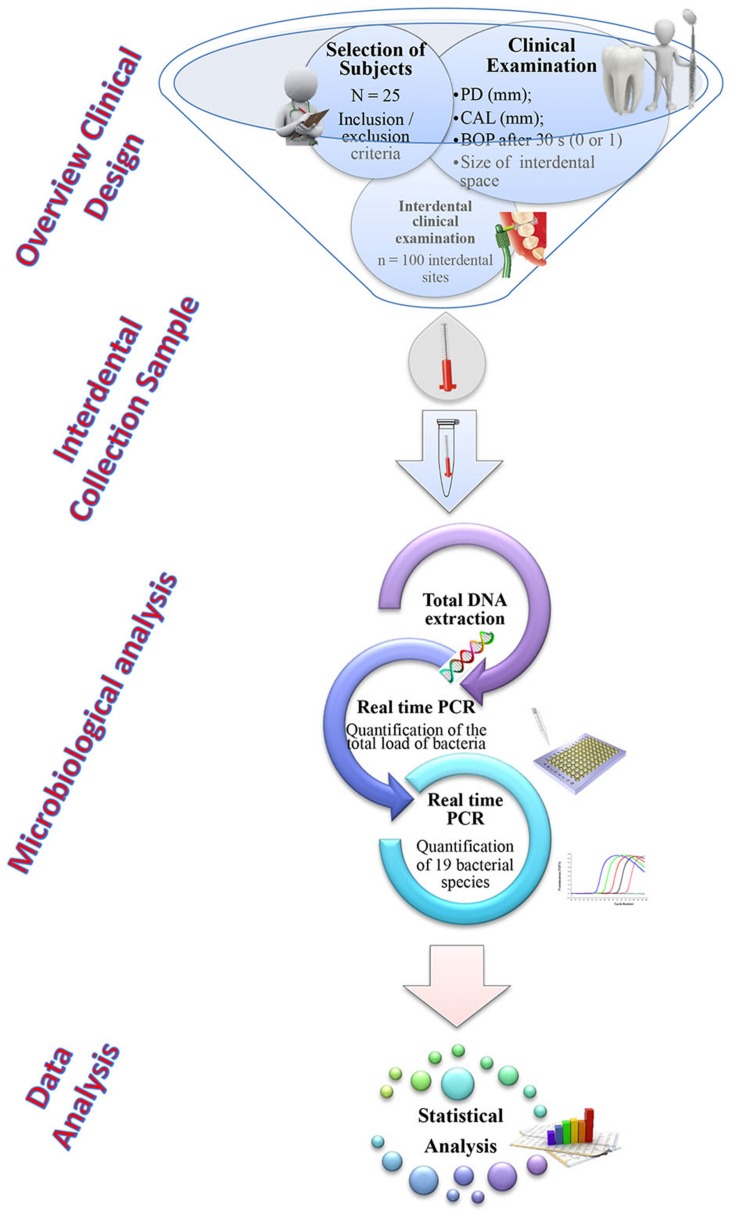
**Workflow of the experiment**.

The workflow of the experiment was described in **Figure 1**.

### Subject Population

Twenty-five Caucasian subjects diagnosed as periodontally healthy (H) were recruited between January and April 2015 from a pool of first-time volunteers who were referred to the Department of Public Health of the Faculty of Oral Medicine at the University of Lyon (UCBL), France. Written informed consent was obtained from all enrolled individuals in accordance with the Declaration of Helsinki. The study protocol was reviewed and approved by the National Ethics Committee and by the National Commission of Informatics and Liberties, France.

The inclusion criteria were: (i) age 20–35 years-old, (ii) good general health, and not pregnant or breastfeeding, (iii) no health conditions that required antibiotic prophylaxis before interproximal probing, (iv) periodontally healthy, (v) tooth brushing at least twice per day, (vi) no experience with interdental cleaning – interdental brushing or dental flossing, (vii) no intake of systemic antimicrobials during the previous 6 months, (viii) no use of chlorhexidine or over-the-counter mouthwash, (ix) no implants or orthodontic appliances, (x) no previous periodontal illness or treatment, (xi) the presence of at least 24 natural teeth, (xii) the presence of four premolar-molar pairs, (xii) non-smokers, and (xiii) a willingness to return 3 weeks after the clinical investigation for microbiological tests.

The clinical inclusion criteria for each premolar-molar interdental site were: (i) accessibility of the interdental space for the four sites (15–16, 25–26, 35–36, and 45–46) by the interdental brush in each subject; (ii) no interproximal caries or dental or prosthetic restorations; (iii) no interdental diastema; (iv) no clinical signs of inflammation, such as redness, swelling, or bleeding on probing (BOP) after 30 s; (v) no pocket depth (PD) > 3 mm or clinical attachment loss (CAL) > 3 mm; and (iv) the subjects were judged to be free of gingivitis or periodontitis.

The exclusion criteria were: (i) teeth missing due to periodontal reasons, (ii) having any other concomitant systemic disorder, (iii) having diseases affecting the immune system, (iv) receiving medication, such as anti-platelet or anti-coagulant agents, (v) having a professional prophylaxis 4 weeks prior to the baseline examination, (vi) having a history of periodontal disease or treatment, and (vii) subjects undergoing a course of dental or orthodontic treatment.

### Classification of Subjects as Periodontally Healthy

All subjects were diagnosed according to criteria described by the American Academy of Periodontology (Armitage, 1999), with some modifications (da Silva-Boghossian et al., 2011). The H subjects presented ≤ 10% of sites with BOP after 30 s and/or overt gingival redness, but no PD (distance from free gingival margin to the bottom of the sulcus) or PD ≤ 3 mm or CAL > 3 mm.

### Calibration of Examiners

In a group of 10 individuals who did not participate in this study, pairs of examinations were conducted in each individual, with a 1-h interval between them. Intraclass correlation coefficients for PD and CAL were calculated at the site level. The intra- and inter-examiner coefficients for CAL ranged between 0.80 and 0.85, and between 0.75 and 0.85 for PD.

### Clinical Examination

Standardized clinical monitoring was performed 3 weeks before microbiological monitoring. The subjects were submitted to a medical/dental anamnesis, and information regarding their age, gender, and smoking status was obtained. The clinical examination was performed by trained and calibrated periodontists. Clinical measurements were taken at six sites per tooth (mesio-buccal, buccal, disto-buccal, disto-lingual, lingual, and mesio-lingual) on all teeth, with the exception of the third molars, as previously described (Haffajee et al., 1983). The clinical parameters were measured in the following order: (i) PD (mm), (ii) CAL (mm), and (iii) BOP after 30 s (0 or 1). The full-mouth clinical measurements included BOP, PD and CAL, which were recorded using a North Carolina periodontal probe (Hu-Friedy, Chicago, IL, USA). Clinical assessments of the interdental spaces were performed using an IAP CURAPROX© colorimetric probe (Curaden, Kriens, Switzerland) and registered the diameter of all the interproximal spaces of four pairs of teeth (premolar-molar). At the end of the examination visit, the participants were instructed to brush their teeth 3 h before the sampling visit and not to drink, eat or practice oral hygiene during this period.

### Interdental Sample Collection

For all subjects, the same four interdental sites (15–16, 25–26, 35–36, and 45–46) were assessed (total 100 sites). The appropriate prime interdental brushes (Curaden, Kriens, Switzerland) were selected based on the clinical assessment of the interdental spaces (Bourgeois et al., 2015). Each previously selected tooth was isolated with sterile cotton rolls and the interdental biofilm was removed with a sterile, calibrated interdental brush (IDB). For each sample, the IDBs were placed in 1.5 mL sterile microcentrifuge tubes and stored at 4°C for further processing.

### Microbiological Analysis

#### Total DNA Extraction

Total DNA was isolated from the interdental brushes using the QIAcube^®^ HT Plasticware and Cador^®^ Pathogen 96 QIAcube^®^ HT Kit (Qiagen, Hilden, Germany), according to manufacturer’s guidelines. The elution volume used in this study was 150 μL. DNA quality and quantities were measured using an ultraviolet spectrophotometer at 260 and 280 nm.

#### Quantitative Real-Time PCR Assays

To quantify the total bacterial load (TB) and that of 19 pathogen species (*A. actinomycetemcomitans*, *A. odontolyticus, A. viscosus, C. concisus, C. gracilis, C. ochracea, C. rectus, C. sputigena, E. corrodens, F. nuc, P. gingivalis, P. intermedia*, *P. micra*, *P. nigrescens*, *S. mitis, Streptococcus spp., T. forsythia, T. denticola, V. parvula)* present in the biofilm interdental samples, qPCR was undertaken using universal primers for the 16S rRNA genes and species-specific primer sets.

Simplex quantitative real-time PCR assays were performed in a 10 μL reaction composed of 1× SYBR^®^ Premix Ex Taq^TM^ Tli RNaseH Plus (TaKaRa, Shiga, Japan), 2 μL of the extracted DNA and 1 μM of each primer. The bacterial primers used are derived from previously published ribosomal 16S sequences and have been adapted to the real-time PCR conditions (**Table 1**). These PCR primers were manufactured by Metabion International AG (Planegg, Germany).

**Table 1:** Species-specific and ubiquitous real-time PCR primers for 19 periodontal bacteria, the annealing temperature, and the limit of quantification.

Target	Primer pairs (5′–3′)	Reference	Annealing temp (°C)	LOQ (E+02)
TB	CCATGAAGTCGGAATCGCTAGT GCTTGACGGGCGTGTG	Kozarov et al., 2006	66	200
*Aa*	AAACCCATCTCTGAGTTCTTCTTC ATGCCAACTTGACGTTAAAT	Kobayashi et al., 2008	60	10
*Pg*	AGGCAGCTTGCCATACTGCG ACTGTTAGCAACTACCGATGT	Sakamoto et al., 2001	60	4
*Tf*	GCGTATGTAACCTGCCCGCA TGCTTCAGTGTCAGTTATACCT	Sakamoto et al., 2001	60	80
*Td*	TAATACCGAATGTGCTCATTTACAT TCAAAGAAGCATTCCCTCTTCTTCTTA	Sakamoto et al., 2001	60	10
*Pi*	CGTGGACCAAAGATTCATCGGTGG ACCGCTTTACTCCCCAACAAA	Fouad et al., 2002	60	60
*Pm*	AGAGTTTGATCCTGGCTCAG ATATCATGCGATTCTGTGGTCTC	Fouad et al., 2002	60	60
*Fn*	AGAGTTTGATCCTGGCTCAG GTCATCGTGCACACAGAATTGCTG	Fouad et al., 2002	60	40
*Cr*	TTTCGGAGCGTAAACTCCTTTTC TTTCTGCAAGCAGACACTCTT	Kobayashi et al., 2008	60	20
*Ec*	GGGAAGAAAAGGGAAGTGCT TCTTCAGGTACCGTCAGCAAAA	Kozarov et al., 2006	60	50
*Pn*	ATGAAACAAAGGTTTTCCGGTAAG CCCACGTCTCTGTGGGCTGCGA	Fouad et al., 2002	66	5
*Cg*	AGAGTTTGATCCTGGCTCAG GGACGCATGCCCATCTTTCACCACCGC	Kobayashi et al., 2008	66	5
*Co*	AGAGTTTGATCCTGGCTCAG GATGCCGCTCCTATATACTATGGGG	Kobayashiet al., 2008	66	5
*Cs*	AGAGTTTGATCCTGGCTCAG GATGCCGCTCCTATATACCATTAGG	Kobayashi et al., 2008	66	5
*Cc*	GGCTCAAAAGAGATCGCTCA CCCTCAACAACGCTTAGCTC	Chaban et al., 2009	66	5
*Smitis*	GAGTCCTGCATCAGCCAAGAG GGATCCACCTTTTCTGCTTGAC	Suzuki et al., 2005	66	5
*Sspp*	AGAGTTTGATCCTGGCTCAG GTACCGTCACAGTATGAACTTTCC	Fouad et al., 2002	66	10
*Ao*	CTTTGGGATAACGCCGGGAAAC CTACCCGTCAAAGCCTTGGT	Yang et al., 2007	66	5
*Av*	ATGTGGGTCTGACCTGCTGC CAAAGTCGATCACGCTCCG	Suzuki et al., 2005	60	5
*Vp*	GAAGCATTGGAAGCGAAAGTTTCG GTGTAACAAGG-GAGTACGGACC	Igarashi et al., 2009	60	5

*LOQ, limit of quantification; TB, total bacterial count.*

The assays were performed on the Rotor-Gene^®^ Q thermal cycling system (Qiagen, Hilden, Germany) with the following program: 95°C for 30 s, followed by 40 cycles of 10 s at 95°C, 10 s at the appropriate annealing temperature (**Table 1**), and 35 s at 72°C. For the total bacterial load and that of all species, a final melting curve analysis (70–95°C in 1°C steps at 5 s increments) was performed. Fluorescence signals were measured every cycle at the end of the extension step and continuously during the melting curve analysis. The resulting data were analyzed using Rotor-Gene Q Series software (Qiagen, Hilden, Germany).

Serial dilutions of a bacterial standard DNA provided by Institut Clinident SAS (Aix en Provence, France) were used in each reaction as external standards for the absolute quantification of the targeted bacterial pathogens. The standard bacterial strains used for standard DNA production came from DSMZ (Germany), CIP Collection of Institut Pasteur or from BCMM/LMG Bacteria Collection: *Aa* (DSM No. 8324), *Pg* (DSM No. 20709), *Tf* (CIP No. 105220), *Td* (DSM No. 14222), *Pi* (DSM No. 20706), *Pm* (DSM No. 20468), *Fn* (DSM No. 20482), *Cr* (LMG No. 7613), *Ec* (DSM No. 8340), *Pn* (DSM No. 13386), *Cg* (DSM No. 19528), *Co* (DSM No. 7271), *Cs* (DSM No. 7273), *Cc* (DSM No. 9716), *Smitis* (DSM No. 12643), *Sspp* (*S. mitis* DSM No. 12643), *Ao* (DSM No. 43760), *Av* (DSM No. 43327), and *Vp.* (CIP No. 60.1). The limit of quantification (LOQ) of the method is summarized in **Table 1**.

### Statistical Analysis

The statistical analysis consists of three main steps, namely producing descriptive summaries of the data, modeling the data using a mixed (linear) model and assessing the correlations between bacterial abundances. Prior to these steps, we transformed the original count data to handle missing data points, namely the measurements that fell under the quantification threshold (LOQ) of the quantitative real-time PCR device. The missing values for a given species were replaced by half of the corresponding quantification thresholds given in **Table 1**. We performed simulations to ensure that this simple strategy provided a reasonable estimation of the mean and standard deviation of the original count distribution. To test for potential effects of sex, age, interdental space and the location of each site, we used a mixed linear model for the log-count abundance of each species at a measured site. This model includes two categorical variables as fixed effects (sex and mouth location), two numerical variables as fixed effects (age and interdental space) and one categorical variable as a random effect (subject). This random effect was introduced for a subject to model the correlation between the four sites of a given subject. Each coefficient in the regression was tested against the null hypothesis, which indicates that the coefficient is zero using a likelihood ratio test, and we reported that p-values less than 0.05, 0.01, and 0.001 were low, medium, and strong evidence against the null hypothesis, respectively. To perform the correlation analysis, we used the residuals of the model described above to avoid over-estimating the inter-site correlation (sites from the same patient are positively correlated, and we observed that fixed effects can also induce a correlation among sites). The trees associated to the correlation plot were obtained by hierarchical clustering with complete linkage.

All statistical analyses and associated plots were performed using the R environment (R Core Team, 2015), specifically the lme4 package (Bates et al., 2015), to estimate the mixed model. The R scripts we developed are provided in **Supplementary Data 1**.

## Results

### Age, Sex, and Clinical Characteristics of the Study Group

The age, the sex and clinical assessments of the study group are summarized in **Table 2**. The sample consisted of 25 subjects (10 females and 15 males) with a mean age of 26.8 ± 4.6 years. The mean number of teeth present is 28.9 ± 1.2. Missing teeth are due to orthodontic extractions (3%) and absence of the third molars (97%). The clinical periodontal conditions of the subjects are indicated by the mean values of BOP (0.16 ± 0.08), PD (0.95 ± 0.21), and CAL (0.95 ± 0.21). At the sampling site level, the mean value of PD is 1.40 ± 0.21 and the mean CAL is 1.54 ± 0.24. Globally, 55% of the 100 interdental spaces had a small diameter of 0.7 mm, and 25% had a diameter of 0.8 mm (**Table 3**).

**Table 2:** Age, sex, and characteristics of the full mouth and sampled sites of the study group.

**Subjects**	
Age (years)	26.8 ± 4.6
Sex	
Male	15
Female	10
Teeth	28.9 ± 1.2
**Full mouth**	
BOP (%)	0.16 ± 0.08
PD (mm)	0.95 ± 0.21
CAL (mm)	0.95 ± 0.21
**Sampled sites**	
BOP (%)	0.00 ± 0.00
PD (mm)	1.40 ± 0.21
CAL (mm)	1.54 ± 0.24

*The values are mean ± standard deviation, and the numbers of subjects are indicated. BOP, bleeding on probing; PD, pocket depth; CAL, clinical attachment level.*

**Table 3:** Average abundances for species of the Socransky complexes in various subgroups of the cohort.

Variable	*n*	TB	TS	*Av*	*Vp*	*Ao*	*Ec*	*Cs*	*Co*	*Cc*	*Aa*	*Smitis*	*Sspp*	*Cg*	*Cr*	*Pi*	*Pn*	*Pm*	*Fn*	*Pg*	*Tf*	*Td*
All^a^	100	10	7.9	2.6	5.6	4.4	6.3	5	5.8	3.5	2.9	5	6.2	4.8	6.6	6.2	4.2	5.5	7.5	3	6	4.6
Bacteria/TB^b^	100	100	0.94	0.00	0.02	0.00	0.10	0.01	0.03	0.00	0.00	0.00	0.03	0.00	0.15	0.09	0.00	0.02	0.37	0.02	0.02	0.04
Positives sites^b^	100	100	100	22	100	92	100	100	97	87	6	100	100	98	96	81	87	88	100	19	93	49

Age (years)			*				*			*					*						**	*
20–24^a^	44	9.9	7.8	2.6	5.7	4.5	6.2	5	5.7	3.3	2.9	5.2	6.3	4.7	6.4	6.1	4	5.3	7.5	2.6	5.5	4
25–29^a^	24	9.9	7.7	2.7	5.6	3.9	5.9	4.1	5.8	3.1	2.7	4.8	5.9	4.6	5.8	5.7	4	5.2	7.3	3.5	6	3.9
30–35^a^	32	10.2	8.3	2.5	5.5	4.6	6.9	5.5	5.9	4.1	3.1	5	6.2	5.2	7.4	6.7	4.7	6.1	7.7	3.2	6.6	5.9

Sex								*						*								
Male^a^	60	10	7.9	2.5	5.6	4.3	6.4	5.1	5.9	3.5	2.8	5.1	6.2	4.6	6.7	6	4	5.6	7.4	2.9	5.8	4.7
Female^a^	40	10.1	8	2.7	5.6	4.5	6.3	4.8	5.7	3.6	3	4.9	6.2	5.2	6.5	6.5	4.5	5.5	7.6	3.2	6.2	4.4

Arcade							***	*										*				
Upper^a^	50	10	7.9	2.6	5.6	4.4	6.7	5.1	5.9	3.6	2.8	5.1	6.2	4.9	6.5	6	4.2	5.4	7.5	3	5.9	4.4
Lower^a^	50	10	7.9	2.6	5.6	4.4	6	4.8	5.7	3.4	3	5	6.2	4.8	6.7	6.4	4.2	5.7	7.5	3	6	4.8

IDB size			*				*			*					*						**	*
0.6 mm^a^	5	9.9	7.8	2.4	6	4.5	6.2	4.4	5.7	3.9	2.7	4.4	5.9	4.7	6.7	6.2	3.9	5.6	7.4	2.6	6.4	4.6
0.7 mm^a^	55	10	7.8	2.6	5.7	4.3	6.1	4.6	5.8	3.1	2.8	4.8	6	4.8	6.2	6	4.1	5.5	7.4	2.8	5.8	4.2
0.8 mm^a^	25	10.1	8	2.6	5.6	4.4	6.5	5.3	5.9	3.7	2.8	5.4	6.4	4.9	7	6.2	4.4	5.5	7.6	3	6.1	5.2
0.9 mm^a^	8	10.3	8.3	2.6	5.4	4.2	7.2	6.2	5.9	4.4	2.7	4.9	6.3	5.2	7.3	6.6	4.6	5.9	7.6	3	6.3	5.7
1.1 mm^a^	7	10.3	8.2	2.4	5.3	5.1	6.6	6	5.2	4.5	4.2	5.7	6.8	4.9	7.2	7	4.4	5.8	7.7	4.6	6.1	4.7

The first three columns correspond to the size of the subgroup, the mean abundance of the total bacterial load and the mean abundance of species from the Socransky complexes only. The other columns correspond to the mean abundance of each bacterial species. The line “Bacteria/TB” represents the proportion of each bacterial species or groups of bacteria (TS) with respect to the total bacterial load (TS). The colors refer to the colors of the Socransky complexes. The line “Positives sites” analyzes the proportion of sites expressing one bacterial species or a group of bacteria (TS) with respect to the ID sites studied. Systematic significance tests were performed between the bacterial species abundances and the tested experimental factors (age, sex, arcade, and IDB size, see Materials and Methods). The stars indicate the test results: low level of significance (*p < 0.05), medium level of significance (**p < 0.01), high level of significance (***p < 0.001). ^a^mean log-count; ^b^percentage; n, number of interdental sites; TB, total bacterial load; TS, total Socransky.

### Total Genome Count and Socransky Genome Count

Because the counts for bacterial abundance may span several orders of magnitude from one site to another (**Supplementary Table S1**), it is more appropriate to use log_10_-scale units and consider the means of the log_10_-counts. Hence, in the subsequent sections, averages should be understood to report log-averages (i.e., geometric means).

The mean counts for the total bacterial load (TB) and that of the 19 evaluated species in the interdental biofilm are reported in **Table 3**. An average of approximately 10^10^ bacteria was collected in one interdental space, and the pool of 19 species, named the total of Socransky (TS), represented 10^7.9^ bacteria. No significant effects of the age, sex, arcade or ID space diameter factors on the total bacterial load were observed. However, the pooled levels of the 19 species were significantly influenced by age and the diameters of the ID spaces (*p* < 0.05).

### Individual Genome Count

**FIGURE 2:**
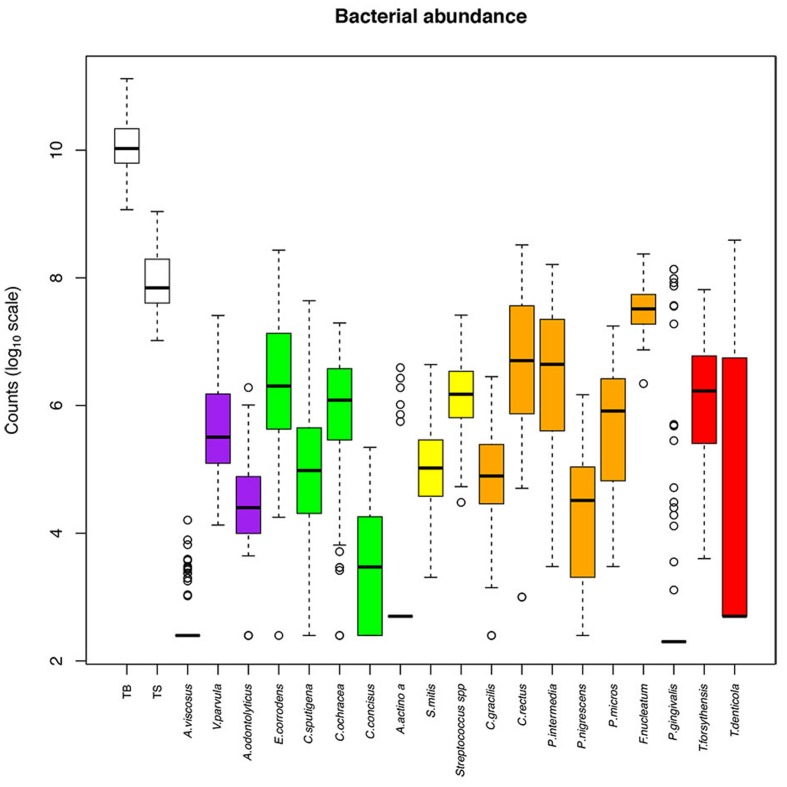
**Abundance of bacterial species among the interdental sites**. The counts are reported on a log_10_ scale. Each box represents the first quartile, median quartile, and third quartile, from bottom to top. The first box on the left (TB) corresponds to the total bacteria and the second (TS) corresponds to the subtotals for species of the Socransky complexes. The colors in boxes refer to the colors of the Socransky complexes. TB, total bacterial load; TS, total Socransky.

The abundance of the 19 evaluated species among the samples is presented in **Figure 2**, and the relative abundance is presented in **Supplementary Figure S1**. The bacteria we tested were expressed at various levels. *F. nucleatum* was the most abundant species (10^7.5^ bacteria in one ID space, 0.37% of TB), whereas the least abundant species were *A. viscosus* (10^2.6^ bacteria in one ID space, less than 0.01% of TB), *A. actinomycetemcomitans* (10^2.9^ bacteria in one ID space, less than 0.01% of TB) and *P. gingivalis* (10^3^ bacteria in one ID space, 0.02% of TB; **Table 3**).

Within each Socransky complex, the expression level of each bacterium differed. The main bacteria were: *V. parvula* for the purple complex, *E. corrodens* for the green complex, *Streptococcus sp.* for the yellow complex, *F. nucleatum* for the orange complex and *T. forsythensis* for the red complex. *P. gingivalis* was detected (number of bacteria > LOQ) in only 19% of the tested sites, which explains why the median, lower and upper quartiles are superimposed. The same occurs for *A. viscosus* (22% of sites) and *A. actinomycetemcomitans* (6% of sites; **Table 3**).

The genome counts for *E. corrodens, C. concisus, C. rectus, T. denticola*, and *T. forsythensis* increased significantly with subject age (**Table 3**). They were also significantly influenced by the diameter of the ID space for the same bacteria. There was no significant evidence for an effect on the other bacteria tested in our setting. *C. sputigena* was significantly higher in males than in females, whereas *C. gracilis* was significantly higher in females than in males. No significant differences according to arcade were observed, except for *E. corrodens* and *C. sputigena*, which were increased in the upper arcade, and *P. micros*, which was increased in the lower arcade.

### Socransky Complexes

**FIGURE 3:**
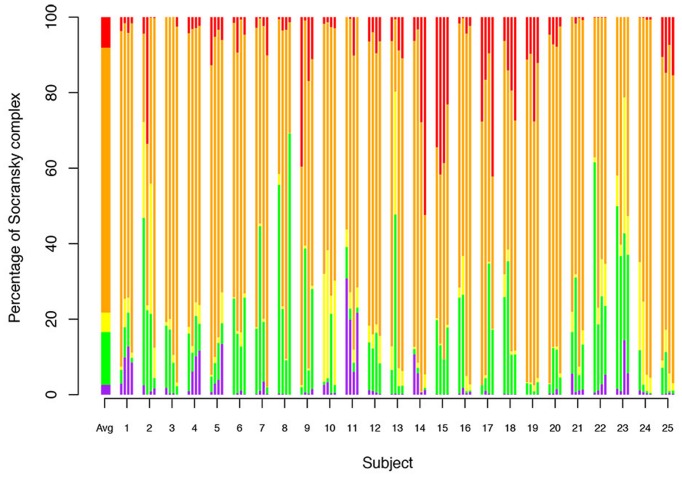
**Relative abundance of Socransky complexes among the subjects.** Percentage of Socransky complex = ΣCounts of the bacteria for one Socransky complex/ΣCounts of the 19 bacteria of the Socransky complexes. The first bar displays the average proportion of each complex in the population. The other bars display the average proportion of each complex in one site. Each subject corresponds to a group of four stacked bars (one for each measured site). The colors refer to the colors of the Socransky complexes: Blue = *Av*, Pink = *Vp*+ *Ao*, Green = *Ec*+ *Cs*+ *Co*+ *Cc*+ *Aa*, Yellow = *Smitis*+ *Sspp*, Orange = *Cg*+ *Cr*+ *Pi*+ *Pn*+ *Pm*+ *Fn*, and Red = *Pg*+ *Tf*+ *Td*. Avg: Average.

The 19 bacteria studied here were grouped into the complexes defined by Socransky et al. (1998). The percentages of each of these complexes per site were analyzed (**Figure 3**). The main complex was the orange complex, which represents an average of 70.17%, followed by the red complex (8.08%), the green complex (13.97%), the yellow complex (5.17%), the purple complex (2.61%) and the blue complex (<0.01%).

**FIGURE 4:**
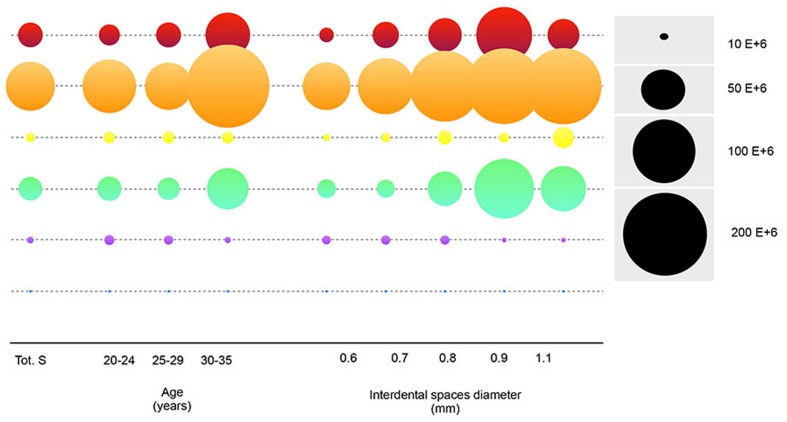
**Mean counts of the Socransky complexes according to age and interdental diameter.** The disk surface is proportional to average of the bacterial counts. The colors of the boxes refer to classes of Socransky complexes. Blue = *Av*, Pink = *Vp*+ *Ao*, Green = *Ec*+ *Cs*+ *Co*+ *Cc*+ *Aa*, Yellow = *Smitis*+ *Sspp*, Orange = *Cg*+ *Cr*+ *Pi*+ *Pn*+ *Pm*+ *Fn*, and Red = *Pg*+ *Tf*+ *Td*. Avg: Average.

The comparison of the mean value of each Socransky complex according to age and the interdental space diameters was shown in **Figure 4**. There was a strong increase in the green, orange and red complexes after 30 years of age. According to the size of the interdental spaces, increases in the abundance of the green complex and the orange and red complexes were observed for diameters ranging from 0.6 to 0.9 mm. Then, their abundances decreased for the 1.1 mm diameter.

**FIGURE 5:**
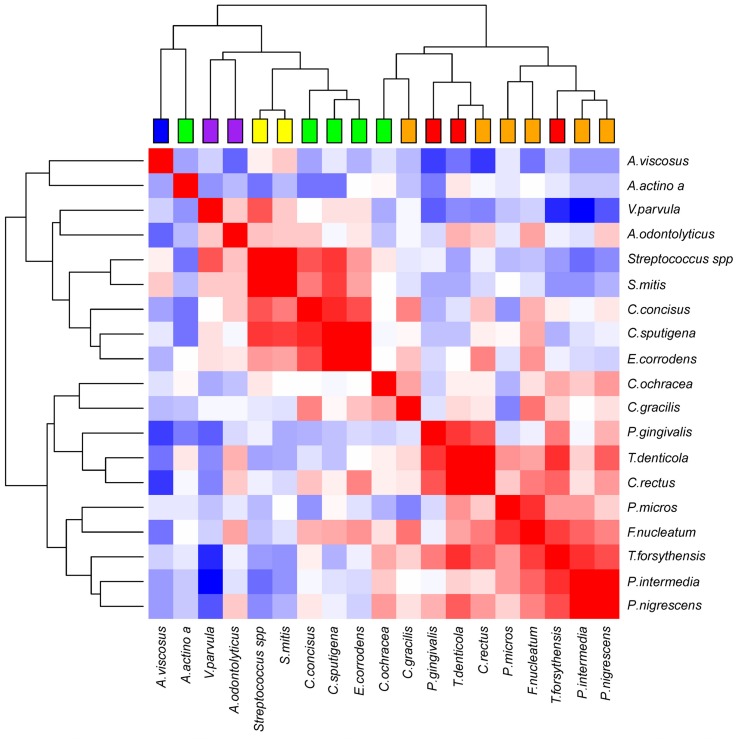
**Correlation plot of the abundances of the bacterial species, corrected for age, interdental space and individual-specific effects.** The red, white, blue squares indicate positive, zero, negative correlations, respectively. The colored leaves on the top dendrogram represent the six Socransky complexes.

**Figure 5** shows the correlations between our 19 bacterial species and the 100 measured ID sites, after subtraction of the inter-site correlations and removal of the fixed effects related to age and interdental space. Even after these corrections, the matrix still shows a strong correlation structure, which highlights several groups (clusters, even) of correlated species. Strikingly, those groups are in close concordance with the Socransky complexes; species from the red and orange complexes cluster together, whereas species from the yellow, purple, and green complexes form three distinct clusters. Two species of the green complex seem less correlated with the group. Although, in the case of *A. actinomycetemcomitans*, there are only six interdental sites where the bacteria passed the quantification threshold, which undoubtedly makes its placement on the dendrogram much less reliable.

## Discussion

To our knowledge, this investigation is by far the largest study employing real-time PCR to study periodontal pathogens in healthy interdental plaques. An understanding of the mechanisms involved in the onset and progression of periodontal diseases could greatly help establish effective ways to prevent and treat of these diseases and decrease the risk factors for relevant systemic disorders. In oral health, the interdental space is a very specific location. From an anatomical point of view, it is not easily accessible to brushing. From a physiological point of view, it is the seat of many more or less virulent bacteria; it is not only the location where interproximal caries are initiated but also the location of periodontal diseases, such as gingivitis and periodontitis.

Gingivitis is an inflammation of the periodontal marginal tissue in response to bacterial biofilms that adhere to tooth surfaces (Socransky, 1977). Therefore, the pathogenesis has been separated into the initial, early, and established stages, each with characteristic features. Several factors have hindered investigations into the etiology of gingivitis (Tatakis and Trombelli, 2004). An individual variation in the gingival inflammatory response to the dental biofilm has been reported (Trombelli and Farina, 2013). Clinical manifestations of gingivitis are episodic phenomena characterized by discontinuous bursts of acute inflammation. In natural human populations, gingivitis symptoms can be reversible and volatile because numerous internally or externally imposed disturbances, including oral hygiene practices (personal or professional), impairment of the immune system, injury, diet and the oral state, may all potentially affect disease development and confound disease monitoring (van der Weijden et al., 2002; Sharma et al., 2004; Trombelli and Farina, 2013). Most lesions are transient or persistent but not progressive. The gingivitis microflora differs in children, adolescents, young adults and adults (Wu et al., 2016). As a worldwide health concern, it affects most children and adolescents (Petersen et al., 2005; Jin et al., 2011).

The clinical signs of gingivitis either do not appear as plaque accumulates, or they are greatly delayed in children, and the inflammatory infiltrate mainly consists of T lymphocytes. The conversion to a B cell lesion does not appear to occur (Page, 1986). Moreover, the current clinical diagnoses of gingivitis are typically based on individual observations and judgment by human examiners and the results between patients and examiners can be difficult to compare (Huang et al., 2014). This raises the question of what level of plaque will result in clinically significant gingivitis. Unfortunately, at present, there is no evidence on which to base such a threshold for either gingivitis or plaque (Imrey et al., 1994). Page and Schroeder (1976) reported that the initial changes from health to plaque-induced gingivitis are not detectable in the clinic. Overall, their conclusions are still largely accepted today (Lang et al., 2009).

The anatomical characteristics of the periodontium, such as gingival thickness, gingival width and alveolar bone morphology, will determine periodontium behavior when it is submitted to physical, chemical, or bacterial injury (Malhotra et al., 2014). The periodontium is mainly the interdental spaces between the molars and premolars, which are susceptible to gingival inflammation (Kassebaum et al., 2014; Eke et al., 2015).

In this variably sized space, the interdental papilla is only lightly keratinized and is thus more permeable to bacterial products – lipopolysaccharides (endotoxins), chemotactic peptides, protein toxins, and organic acids – released by the biofilm (Bosshardt and Selvig, 1997; Scannapieco, 2004). The gingival epithelium, which is exposed to the buccal microflora, protects the underlying tissue by maintaining a periodontal homeostasis (Damek-Poprawa et al., 2013). To achieve this, the keratinocytes possess Pattern Recognition-Receptors capable of specifically recognizing different bacterial sequence patterns. The cellular information transmitted through these receptors induces the innate immune response, particularly the production of antimicrobial peptides and mediators of inflammation (Blumenthal et al., 2006). In some cases, this response is insufficient, which may lead to gingivitis or periodontitis (Honda et al., 2006).

Bacteria circulating in the saliva will gradually coat the enamel surface to form the biofilm. Unlike the enamel lingual and palatal surfaces, which undergo self-cleaning by the action of the tongue, or buccal surfaces that undergo self-cleaning by alveolar mucosa, the interdental space has no self-cleaning mechanism, and the passage of salivary fluid has little or no action on the formation of the interproximal biofilm (Dentino et al., 2013).

Furthermore, in daily oral hygiene activities, it is technically impossible to reach this space and to disrupt the biofilm of the healthy adult in the posterior parts of the mouth (Poklepovic et al., 2013). The composition of the interdental biofilm has never been studied, but it has been investigated for the extent of its control by the use of brushing techniques (IDB and flossing; Sambunjak et al., 2011). It is often considered a supragingival biofilm. In fact, due to its anatomical location between two teeth and the gum, the bacteria are in a more anaerobic environment than the supragingival biofilm located on the lingual, palatal and vestibular surfaces Cafiero and Matarasso, 2013). Some of these factors should be an explanation for why the bacterial abundance of the red, orange, and green complexes decreases as the interdental size reaches 1.1 mm – the largest diameter – in our study. A larger interdental space could lead to salivary cleaning and a highly aerobic environment, and perhaps a cleaning related to the anatomy of the tongue would be able to modify nutrients (Mariotti and Hefti, 2015).

Our study characterizes the interdental biofilm of periodontally healthy young adults. For this, a new technique was used to collect the biofilm that consists of using IDB. Many studies have employed different collection techniques, including paper points, sterile cotton swaps, curettes or strips, in the sulcus, mesial and distal surfaces of teeth and were mainly focused on the subgingival plaque (Teles et al., 2007; Wang et al., 2013). The methods used to sample supragingival or subgingival plaque were described by Wardle (1997). Studies have been performed to ascertain the method of sampling that will yield the most organisms (Renvert et al., 1992) and to determine the area from which it is appropriate to take the sample (Katsanoulas et al., 1992). Microscopic observations have shown that these bacteria are mainly localized on the loose surface layer of plaque that is in contact with pocket epithelium (Listgarten, 1976) and have been inserted into the periodontal pocket. The methods focused on approximal (proximal) areas or sites, which are the visible spaces between teeth that are not under the contact area.

The commonly used methods are not suitable for assessing interdental plaque (directly under the contact area) and thereby limit the interpretation of interdental plaque removal. The term interdental refers to the area under and related to the contact point. The interdental gingiva fills the embrasure between two teeth apical to their contact point. It is difficult to clinically assess the middle interdental area, as they are usually not available for direct visualization (Walsh and Heckman, 1985). Even subgingival plaque can be removed because dental floss can be introduced 2–3.5 mm below the tip of the papilla (Waerhaug, 1981). The American Dental Association reports that up to 80% of plaque may be removed with this method (Warren and Chater, 1996; ADA 1984). Based on the available literature on interdental cleaning, the best available data suggest the use of interdental brushes (Schmage et al., 1999; Ishak and Watts, 2007). A meta-analysis showed the superiority of the interdental brush to floss with respect to plaque removal (van der Weijden and Slot, 2011). The sampling technique based on the use of interdental brushing meets the specific aim of our study, which was to collect the biofilm from the interdental space. This new sample collection method is based on two basic principles: (i) the calibrated interdental brush must penetrate the space in an unconstrained manner and (ii) it must completely fill the interdental space with necessary mechanical contact with dental hard surfaces and in the gingival sulcus, as described (**Supplementary Figure S2**).

Our results showed that the interdental space is an area in which biofilm accumulates. In periodontally healthy young adults aged from 20 to 35 years, it contains approximately 10^10^ bacteria, whereas in other studies, such as the study by Field et al. (2012), the interdental space has a total count of 10^7.56^ bacteria for supragingival biofilm in periodontally healthy adults aged from 35 to 56 years.

To collect the best data on the composition of interdental biofilm, real-time PCR was used to study the periodontal pathogens expressed in the interdental biofilm of periodontally healthy sites. It is well-known that periodontal diseases appear when some specific bacteria are expressed and reach a critical level (Socransky and Haffajee, 1992). Therefore, real-time PCR appears to be the best choice to analyze the presence or absence and absolute concentration of 19 parodontopathogens with a high sensitivity and a broad detection range (Boutaga et al., 2005; Smith and Osborn, 2009). Indeed, other microbial detection methods, such as checkerboard DNA–DNA hybridization, immunological assays, and conventional end-point PCR, only provide a qualitative analysis or at best a relative quantification of the target species (Torrungruang et al., 2015).

The 19 bacteria studied here belong to Socransky’s complexes (Haffajee et al., 2004). Indeed, in cases of periodontitis, Socransky’s complexes are the reference, even if it now seems that gingivitis and periodontitis could be caused by a more diverse community of bacteria (Hajishengallis, 2015). Overall, the dendogram we obtained is similar to the results obtained by Socransky et al. (1998), although the two studies are of very different populations of patients. The cohort in Socransky et al. (1998) included patients with or without periodontal syndrome from a very wide age range, but we based our results on a young and healthy population. Ultimately, the association patterns between bacteria species showed a very similar structure. The complexes tended to reflect microbial succession events in developing dental biofilms from early to late colonizers. The early colonizers included members of the yellow complex (some *Streptococcus* species, e.g., *S. mitis* and *Streptococcus spp*.), the green complex (e.g., *A. actinomycetemcomitans*, *E. corrodens*, *C. sputigena*, *C. ochracea, and C. concisus*) and the purple complex (e.g., *V. parvula and A. odontolyticus*). The orange complex bacteria generally appear after the early colonizers are established and include many putative periodontal pathogens, such as *F. nucleatum*, *P. micra*, *P. intermedia*, *P. nigrescens*, *E. nodatum*, and *C. rectus*. The red complex was considered the climax community, and all three of its members (i.e., *P. gingivalis*, *T. forsythia*, and *T. denticola*) are on the list of putative periodontal pathogens (Socransky et al., 1998). In subsequent papers, *Actinomyces* species that are early colonizers were placed in a group called the blue complex (Socransky and Haffajee, 2002, 2005). Importantly, all groups of colonizers, from early to late, contain putative periodontal pathogens (Socransky et al., 1998; Socransky and Haffajee, 2002, 2005). Under appropriate conditions, these bacteria can be important contributors to the initiation and progression of periodontal infections.

Our study highlights the fact that bacteria from the yellow complex (*Streptococcus spp.* and *S. mitis*), the green complex (*E. corrodens, C. gracilis, C. ochracea, C. sputigena*, and *A. actinomycetemcomitans*), the purple complex (*V. parvula* and *A. odontolyticus*) and the blue complex (*A. viscosus*) are correlated. Moreover, they are the first bacteria to constitute the supragingival and subgingival biofilms and are evidence of periodontally healthy patients (Teles et al., 2012; Armitage, 2013). Therefore, the fact that they represent some 18% of the 19 tested bacteria is very surprising because we monitored periodontally healthy subjects. Bacteria of these four complexes are interrelated and each one interacts with the other.

Concerning the yellow complex, *S. mitis* is an early colonizer of the oral biofilm that is found in atherosclerotic plaques of orally healthy subjects and was also associated with endocarditis (Huang et al., 2002; Alkhatib et al., 2012; Eberhard et al., 2016). Our study reveals that it is expressed at levels more than 10-fold higher than *A. actinomycetemcomitans* of the green complex, which could be explained by the fact that streptococci are able to inhibit *A. actinomycetemcomitans* colonization on soft tissue surfaces under flow conditions (Sliepen et al., 2009). Even if the mechanism is not known, *S. mitis* shows prominent inhibitory effects on *A. actinomycetemcomitans* colonization of epithelial cells (Teughels et al., 2007). *S. mitis* also interacts with *P. gingivalis* and inhibits its growth (Tenorio et al., 2011). *Streptococcus spp.* have been found to aggregate with the outer membrane vesicles of *P. gingivalis* (Hiratsuka et al., 2008).

Concerning the blue complex, which is composed of *Actynomyces* species, *A. viscosus* represents some 10^2.6^ bacteria in one ID space. This species co-aggregates with *E. corrodens* from the green complex through the GalNAc-specific lectin (Ebisu et al., 1988), which stimulates the mitogenic activity of B lymphocytes (Nakae et al., 1994).

Concerning the green complex, particularly the three *Campylobacter* species, our study showed that in healthy periodontal subjects, *C. concisus* (10^3.5^ bacteria in one ID space) is expressed at lower levels than *C. ochracea* (10^5.8^ bacteria in one ID space) and *C. sputigena* (10^5^ bacteria in one ID space). In a previous study, Henne et al. (2014) revealed that continuous changes in *Campylobacter* species within and between each microbial complex are observed during periodontitis progression. The authors determined that the proportion of species within the genus *Campylobacter* is a suitable and dynamic marker for periodontitis progression. Based on three bacteria, this theory indicates that the more *C. rectus* and less *C. concisus* are expressed, the more the periodontal disease progresses. *C. gracilis* is associated with the intermediate step. Therefore, our results disagreed with this theory because the subjects were periodontally healthy young adults and the level of expression in decreasing order was *C. rectus*, *C. gracilis* and *C. concisus*.

Among the bacteria of the purple complex, our study revealed that *V. parvula* represented 4x10^5^ bacteria in one ID space. This result agrees with the previous study from Uzel et al. (2011), which showed that the meant count of *V. parvula* in the supragingival biofilm is 3.5 × 10^5^, whereas the mean count in the subgingival biofilm is approximately two-fold lower in periodontally healthy subjects. The same comparison is observed for *A. odontolyticus*. Thus, the ID biofilm will be similar to that of a supragingival biofilm. The function of *V. parvula* has not been elucidated, but it is known to interact with *P. gingivalis* and inhibits its growth (Tenorio et al., 2011).

Although bacteria of the yellow, green, purple, and blue complexes are usually compatible and associated with periodontal health, more and more data tend to prove that these bacteria may be associated with periodontal disease or systemic diseases (López et al., 2011; Abusleme et al., 2013; Chukkapalli et al., 2015; Yost et al., 2015; Chistiakov et al., 2016).

Concerning the orange complex, this study focused on *C. gracilis, C. rectus, F. nucleatum, P. intermedia, P. micra*, and *P. nigrescens.* Throughout the literature, *C. rectus* and *C. gracilis* can be detected at elevated levels in diseased subgingival sites compared with healthy sites (Henne et al., 2014). *C. gracilis* is expressed at higher levels than *C. rectus* in supragingival and subgingival biofilms (Uzel et al., 2011). In contrast, our results show that the expression of *C. rectus* was 100-fold higher than *C. gracilis.*

Our data indicated that *F. nucleatum* is the most abundant species in interdental biofilm, similar to previous reports. Previous studies observed that *Fusobacterium* was the most abundant genus and that *F. nucleatum* is the most abundant species in the subgingival microbiota of periodontally healthy persons (Camelo-Castillo et al., 2015). The role of *F. nucleatum* is currently still controversial. Firstly, *F. nucleatum* is considered an opportunistic pathogen that belongs to the orange complex and is thought to promote dental plaque formation by serving as a bridge bacterium between early- and late-colonizing species of the oral cavity (Settem et al., 2012). Secondly, *F. nucleatum* is considered a commensal or possibly beneficial bacterium due to its ability to induce the production of antimicrobial peptides in gingival epithelial cells (Krisanaprakornkit et al., 2000; Ji et al., 2007). *F. nucleatum* induced Th3 (sIgA)- and Th1 (IFNc and IgG1)-mediated immune responses, whereas *T. denticola* induced a Th1 (IFNc and IgG1)-mediated response. This IFNc-mediated cytokine response was impaired in chronic periodontitis patients, and the Td92-induced IFNc levels were negatively associated with periodontal destruction in patients. These findings may provide new insights into the homeostatic interaction between the immune system and oral bacteria and the pathogenesis of periodontitis (Shin et al., 2013).

*P. micra* (previously known as *Peptostreptococcus*) showed the strongest association with disease and was present at significantly higher absolute and relative numbers in the subgingival biofilm of periodontitis sites (Al-hebshi et al., 2014). There is an increasing amount of evidence on its role as a periodontal pathogen, along with other peptostreptococci (Rams et al., 1992; Kumar et al., 2005; Colombo et al., 2009; Belstrom et al., 2014). However, our study revealed that 88% of the sites of the periodontally healthy subjects expressed *P. micra* with a mean value of 10^5.5^ bacteria, thus questioning the role of this bacterium in periodontal diseases. Moreover, our results led us to hypothesize that this bacterial species could be expressed in the interdental biofilm at higher levels than in the supragingival and subgingival biofilms of periodontally healthy subjects, although our conclusion is not certain because Uzel et al. (2011) used DNA–DNA hybridization and we used real-time PCR to quantify the bacterial counts.

The red complex, which is recognized as comprising the most important pathogens in adult periodontal disease (Suzuki et al., 2013), represents 8.08% of the 19 bacteria analyzed in the interdental biofilm.

In accordance with the study by Griffen et al. (1998), *P. gingivalis* was detected in only 19% of healthy subjects. Our study reveals that *P. gingivalis* represents 0.02% of the interdental biofilm in healthy subjects, suggesting these subjects could develop periodontal disease. Indeed, it has been shown that low levels of *P. gingivalis* (< 0.01% of the total load) were able to induce changes in the composition of the biofilm and cause bone loss in mice (Hajishengallis et al., 2011). Although, it is present at low proportions in the oral microbial community, *P. gingivalis* is considered a ‘keystone’ pathogen that is able to disrupt host–microbe homeostasis through molecular manipulations of selected host-protective mechanisms (Hajishengallis et al., 2011). A large number of studies have shown that *P. gingivalis* modulates innate host defense functions by inhibiting IL-8 secretion, complement activity and TLR4 activation. This inhibition impairs the ability of the host to defend against the oral microbial community at large, resulting in an altered oral flora composition and subsequent inflammatory responses that contribute to the pathogenesis of periodontitis (Darveau et al., 2012). Additionally, the current study indicated that *P. gingivalis* could be correlated with age, as previously described by Matsushita et al. (2015).

The importance of *P. gingivalis* and *T. forsythia* in the initiation of chronic periodontitis and the progression to advanced periodontitis has been reported (van Winkelhoff et al., 2002; Klein and Goncalves, 2003; Yang et al., 2004). In the study of Noda et al. (2007), both *P. gingivalis* and *T. forsythia* were detected in 50% of the gingival tissues obtained from patients with chronic periodontitis at periodontal surgery. In our study, both *P. gingivalis* and *T. forsythia* were detected in 12% of the interdental biofilm of periodontally healthy subjects. This led us to hypothesize that gingivitis or periodontitis could be initiated in these sites.

Chronic periodontitis has a polymicrobial biofilm etiology, and interactions between key bacterial species are strongly implicated in contributing to disease progression. *P. gingivalis*, *T. denticola*, and *T. forsythia* have all been suggested to play roles in disease progression. Of the three species, only *P. gingivalis* and *T. denticola* formed mature, homotypic biofilms, and a strong synergy was observed between *P. gingivalis* and *T. denticola* in polymicrobial biofilm formation (Zhu et al., 2013). Nevertheless, in animal models, a mixed infection with these two organisms has significantly enhanced their virulence potential in abscess formation (Takemoto et al., 1997; Yoneda et al., 2001), suggesting a possible synergistic effect of *P. gingivalis* and *T. forsythia* in periodontal disease. This effect may be due to their binding through proteins on their surface (Zhu and Lee, 2016). Moreover, in a murine experimental model, *P. gingivalis* alone can stimulate the host immune response and induce alveolar bone loss. Its action is potentiated in the presence of *T. denticola*, whereas *T. denticola* alone failed to induce bone loss in this model (Orth et al., 2011). In periodontally healthy patients, our study revealed that these two bacteria are correlated with each other and with *T. forsythia*, indicating that the periodontal disease process could be initiated.

## Conclusion

The interdental biofilm of young periodontally healthy subjects is composed of bacteria that are able to induce periodontitis. The effective presence of the red complex, particularly *P. gingivalis*, a pathogen of heart disease and other systemic diseases, is a strong indicator of the need to develop new methods to disrupt interdental biofilm using daily oral hygiene. Additional studies that are primarily focused on understanding the cellular and molecular pathogenic mechanisms of the red complex and certain other suspected pathogens are necessary.

## Author Contributions

DB and FC conceived the experiments; FC, SV, and JS performed the experiments; PV analyzed the data; and FC, DB, and SV wrote the paper.

## Conflict of Interest Statement

The authors declare that the research was conducted in the absence of any commercial or financial relationships that could be construed as a potential conflict of interest.

## Supplementary Material

TABLE S1 **Bacterial count for the total load of bacteria and for 19 major pathogens in the interdental biofilm**. The table represents the results of 16S qPCR DNA of the healthy donors used in this study. TB, total bacteria; *Aa, A. actino. a; Ao, A. odontolyticus; Av, A. viscosus; Cc, C. concisus; Cg, C. gracilis; Cr, C. rectus; Co, C. ochracea; Cs, C. sputigena; Ec, E. corrodens; Fn, F. nucleatum; Pg, P. gingivalis; Pi, P. intermedia; Pm, P. micra; Pn, P. nigrescens; Td, T. denticola; Tf, T. forsythia; Smitis, S. mitis; Sspp, Streptococcus spp.; Vp, V. parvula*. The colors refer to the colors of the Socransky complexes.

FIGURE S1 **Abundance of bacterial species among samples relative to the total load of bacteria**. Frequencies (Count/Total load of bacteria) are reported in log_10_ scale. Each box represents from bottom to top: first quartile, median, and third quartile. The first box on the left (TS) corresponds to the subtotal for species of the Socransky complex. The colors in boxes refer to the colors of the Socransky complexes.

FIGURE S2 **Calibrated interdental brush in interdental spaces considered clinically healthy. (A)** Top view, **(B)** Front view.

SUPPLEMENTARY DATA 1 **R scripts developed for this study**.
