# Supplementary figures and images for: Quantitative Molecular Detection of 19 Major Pathogens in the Interdental Biofilm of Periodontally Healthy Young Adults
Source: Front Microbiol. 2016 Jun 2;7:840. doi: 10.3389/fmicb.2016.00840 (PMC4889612; doi:10.3389/fmicb.2016.00840)

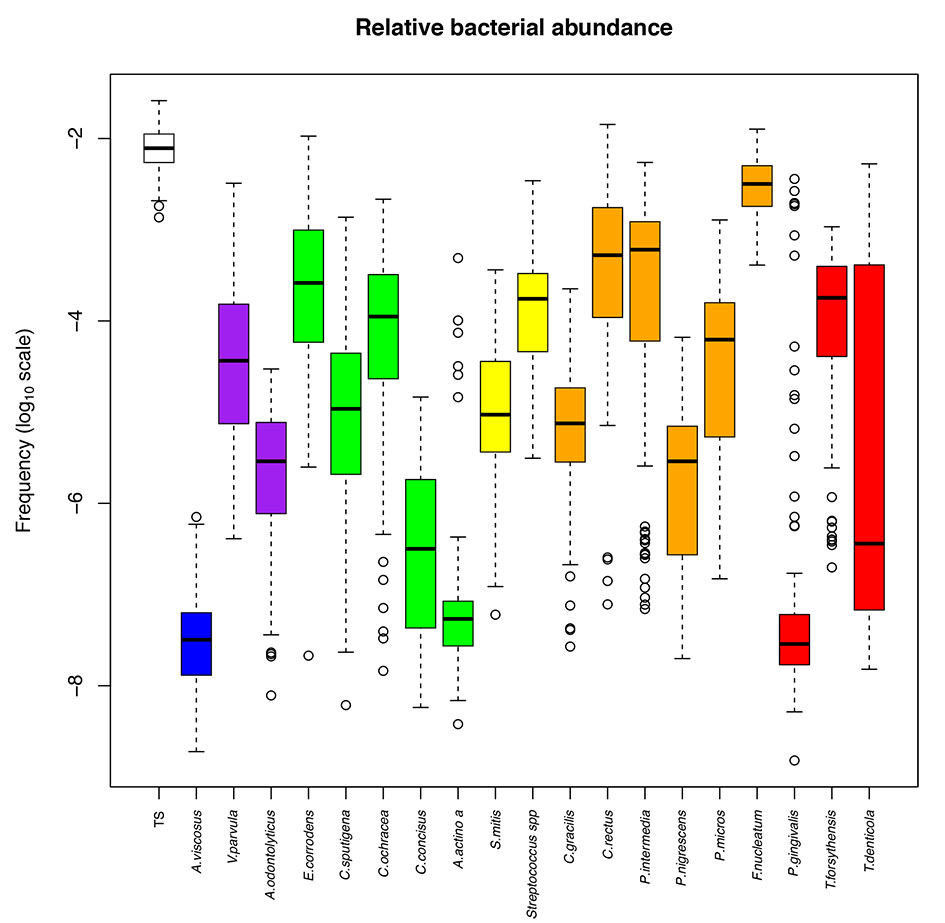

Supplement: FIGURE S1 — Abundance of bacterial species among samples relative to the total load of bacteria. Frequencies (Count/Total load of bacteria) are reported in log10 scale. Each box represents from bottom to top: first quartile, median, and third quartile. The first box on the left (TS) corresponds to the subtotal for species of the Socransky complex. The colors in boxes refer to the colors of the Socransky complexes. [file Image_1.JPEG]

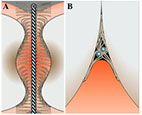

Supplement: FIGURE S2 — Calibrated interdental brush in interdental spaces considered clinically healthy. (A) Top view, (B) Front view. [file Image_2.JPEG]
